# Effects of Surface Crack Shape on Fracture Behavior of Oil Pipelines Based on the MMC Criterion

**DOI:** 10.3390/ma17174406

**Published:** 2024-09-06

**Authors:** Jun Wu, Xiaoyan Gong, He Xue, Rongxin Wang, Zheng Wang

**Affiliations:** School of Mechanical Engineering, Xi’an University of Science and Technology, Xi’an 710054, China; 22205016023@stu.xust.edu.cn (J.W.); gongxymail@163.com (X.G.); 22205016016@stu.xust.edu.cn (R.W.); wangzheng@stu.xust.edu.cn (Z.W.)

**Keywords:** X80 pipeline steel, ductile fracture, MMC criteria, crack shape, location of initiation

## Abstract

This study employs a hybrid numerical-experimental calibration method based on phenomena to determine the fracture parameters of the Modified Mohr–Coulomb (MMC) model. Using a self-developed VUMAT subroutine and the element deletion technique, the fracture process of a wide plate pipeline is thoroughly analyzed. This study investigates the impact of various crack shapes on the fracture response under tensile loading and the influence of surface crack size on the initiation location of a wide plate. These results demonstrate the calibrated MMC fracture model’s accurate prediction of the toughness fracture behavior of X80 pipeline steel. Under equal area conditions of the dangerous section, circular cracks exhibit lower bearing capacity compared to elliptical cracks. Elliptical cracks predominantly propagate in the thickness direction, whereas circular cracks show nearly uniform growth in all directions. Furthermore, when the crack depth is less than half of the wall thickness, the damage accumulation value at the midpoint of the crack front is maximized; conversely, when the crack front is closer to the internal measurement point of the wide plate, the damage accumulation value is maximized.

## 1. Introduction

As of 2022, the total length of operational oil and gas pipelines worldwide has reached approximately 2.02 million kilometers. Natural gas pipelines make up about 1.35 million kilometers, or approximately 67% of the total length. Crude oil pipelines account for roughly 400 thousand kilometers, or about 20% of the total length, while refined oil pipelines contribute approximately 270 thousand kilometers, or about 13% of the total. These pipelines are predominantly concentrated in North America, Europe, Russia, Central Asia, and the Asia-Pacific region [1]. In recent years, as oil and gas exploration has expanded into remote deserts, polar permafrost, and marine regions, high-strength pipeline steels such as X65 to X120 with enhanced strain resistance have been developed [2]. Compared to X65, X80 pipeline steel provides superior yield and tensile strength, allowing for thinner wall thicknesses under the same transport pressure. This results in reduced pipeline weight and lower construction costs [3,4,5]. Pipelines made from X100 and X120 steel grades, which have higher yield and tensile strengths, have also been developed and tested [6]. However, to maximize economic benefits and ensure safety, X80 pipeline steel is widely used for transporting oil and gas both onshore and offshore due to its excellent fracture resistance [7]. During long-distance transportation, pipelines encounter highly intricate environmental conditions, rendering them vulnerable to substantial deformation induced by events such as earthquakes, landslides, and pipeline installation [8,9]. Stress-based design methods are inadequate for situations involving significant strains. To overcome this limitation, an increasing number of researchers are embracing strain-based design [10,11,12,13]. The strain-based design method takes into account the pipe’s ability to de-form under various loads, allowing the pipe to function better than stress-based design methods [14,15,16].

The classification of damage mechanics models into coupled fracture models and non-coupled fracture models depends on whether the influence of damage on the mechanical properties of the material is taken into account. Uncoupled fracture models, with relatively simple expression forms and fewer parameters to be determined, are often used as the basis of judgment in engineering applications [17,18,19]. Bai and Wierzbicki [20] incorporated the impact of the Lode angle on fracture strain, developing the Modified Mohr–Coulomb (MMC) model that considers the dual influence of the Lode parameter and stress triaxiality, which is extensively applied in fracture prediction. Lou [21] employed a physically motivated macroscopic ductile fracture criterion in numerical simulations, predicting the onset of ductile fracture in DP980 steel plates subjected to diverse loading conditions. Han [22] meticulously calibrated the parameters of three models employing a hybrid numerical-experimental approach, subsequently validating the accuracy of the damage fracture model through the use of compact tension (CT) specimens. Tensile testing of full-size pipelines [23,24,25] stands out as the most precise method for evaluating pipeline strain capacity; however, its widespread adoption is limited by the associated high costs. Consequently, wide plate tensile tests, designed to closely mimic actual pipeline size and loading conditions, have emerged as the predominant approach for studying pipeline strain capacity. In contrast to traditional defect assessment methods, the wide plate tensile test demonstrates distinct advantages [26,27], and classic fracture performance specimens for pipeline steel, including single-edged notched tension (SENB) specimens and CT specimens, exhibit a reduction in fracture toughness as the constraint level rises [28]. Wide plate specimens, with larger dimensions compared to small-sized samples, better reflect issues in practical engineering, such as material heterogeneity, the influence of welding residual stress, and the effect of plate thickness on structural performance. Gong et al. [29] formulated a strain-based assessment methodology that correlates the critical strain of the MMC fracture criterion with the tensile strain capacity (TSC) observed in full-sized cracked pipes, enabling a closer alignment with the model proposed by ExxonMobil. Numerical simulations show that the crack shape affects the stability of leaking cracks in pressure pipelines [30,31]. Adjusting the depth of a crack to alter its shape enables a deep-er comprehension of the interplay between crack morphology and the driving force behind propagation, facilitating a more precise evaluation of structural performance in both design and maintenance contexts. Wang et al. [32] conducted a three-dimensional finite element analysis of a semi-elliptical crack in a thick plate, verifying the model by fatigue tests and calculating the J integral at the crack tip based on the crack depth and length, highlighting that the location of the highest J-value is not fixed but depends on the crack’s depth and length. The J-value profile along the crack can exhibit either an inverted U-shape, with a maximum at the deepest point of the crack, or an M-shape, with two symmetrical high J-values either at the surface point of the crack or between the surface point and the deepest point of the crack. Ding et al. [33] proposed a new isotropic uncoupled fracture model. The new model considers the effect of void shape changes and the void coalescence through internal necking on the ductile fracture of metals. It can effectively predict the equivalent fracture strain of AA 2024-T351, AISI 1045, and Q460 aluminum alloy or steel under different stress states. Baral et al. [34] coupled the DF2016 criterion with the non-quadratic anisotropic Yld91 criterion to propose a new anisotropic toughness fracture criterion that represents this enhanced anisotropic fracture location of the material. This new criterion is flexible enough to capture the fracture anisotropy and the fracture formation limit curve of the material. In addition, fracture surfaces plotted in four dimensions for the stress triaxiality and the three main plastic strains showed the loading direction dependence of the fracture location and its sensitivity to the stress triaxiality.

This study examines the fracture behavior of surface crack shapes on X80 pipeline wide plates using the MMC criterion. Initially, the fracture properties of monotonically loaded pull-off specimens of five X80 pipeline steel specimens in different stress states undergo testing, and the parameters in these fracture models are calibrated through finite element analysis. Subsequently, using the calibrated parameters together with the VUMAT subroutine, finite element analysis is performed on the OD 1422 mm × 32.1 mm X80 pipeline wide plate. The investigation explores the influence of various crack shapes and mesh sizes on the tensile performance of pipeline wide plates. In conclusion, using finite element simulations, we analyze the impact of different crack depths on the initiation locations of cracks in pipeline wide plates. This analysis serves as a valuable reference for X80 pipeline engineering failure and structural integrity assessments.

## 2. Overview of the MMC Model

The MMC model is a phenomenological model extensively utilized for elucidating ductile fracture phenomena in diverse materials and under various experimental conditions [35,36,37], where equivalent strain based on stress triaxiality and normalized Lode angle parameters are used to predict crack extension in combination with appropriate post-initiation softening.

In the investigation of ductile fractures, frequently employed parameters for characterization include stress invariance, stress triaxiality, Lode angle, and others. During the plastic deformation stage of materials, the ductile fracture criterion is usually expressed in terms of equivalent fracture strain because the increment of strain is much larger than the increment of stress.

Assuming the material is isotropic [38], the material model can be represented by the three invariants of the stress tensor [*σ*], which are defined by Equations (1)–(3).

(1) $$p=-\sigma_{m}=\frac{1}{3}tr\left[\sigma \right]=-\frac{1}{3}\left(\sigma_{1}+\sigma_{2}+\sigma_{3}\right)$$

(2) $$q=\bar{\sigma }=\sqrt{\frac{1}{2}\left[{\left(\sigma_{1}-\sigma_{2}\right)}^{2}+{\left(\sigma_{2}-\sigma_{3}\right)}^{2}+{\left(\sigma_{3}-\sigma_{1}\right)}^{2}\right]}$$

(3) $$r=\left(\frac{9}{2}\left[S\right]\cdot \left[S\right]:\left[S\right]\right)=\frac{27}{2}\left(\sigma_{1}-\sigma_{m}\right)\left(\sigma_{2}-\sigma_{m}\right)\left(\sigma_{3}-\sigma_{m}\right){]}^{1/3}$$

where [*S*] is the deviatoric stress tensor, which is defined in Equation (4).

(4) [S]=[σ]+p[I]

where [*I*] is the unit tensor, and *σ*_1_, *σ*_2_, and *σ*_3_ are the principal stresses in three directions. The failure strain can be defined as a function of the stress triaxiality *η* and the Lode angle *θ*, with the expression shown in Equation (5).

(5) $$\eta =-\frac{p}{q}=\frac{\sigma_{m}}{\bar{\sigma }}$$

**Figure 1:**
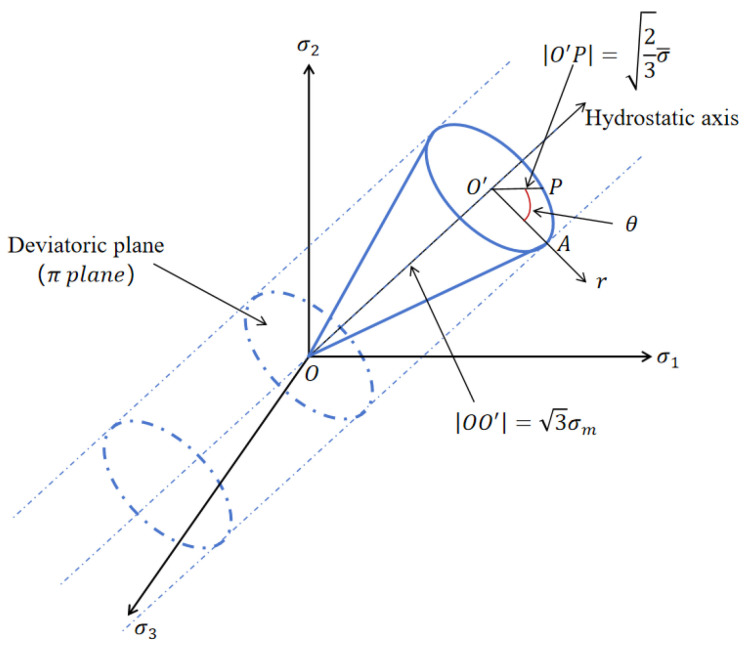
Three coordinate systems in the principal stress space.

The Lode angle parameter is associated with the third deviatoric stress invariant, *ξ*, and Figure 1 illustrates the geometric representation of the Lode angle.

Due to the Lode angle’s range being 0 ≤ θ ≤ *π*/3, the range of *ξ* is −1 ≤ *ξ* ≤ 1. The Lode angle can be normalized as the Lode angle parameter, with $-1\leq \bar{\theta }\leq 1$, expressed in Equation (6).

(6) $$\bar{\theta }=1-\frac{6\theta }{\pi }=1-\frac{2}{\pi }\arccos\xi$$

The Mohr–Coulomb (MC) criterion proposes that fracture occurs when the combination of normal and shear stresses on the fracture surface reaches a critical value. By converting the MC criterion from stress space to strain space, the MMC criterion is formulated. The expression for the MMC criterion is shown in Equation (7).

(7) $$\bar{\varepsilon_{f}}={\left\{\frac{A}{C_{2}}\left[C_{3}+\frac{\sqrt{3}}{2-\sqrt{3}}\left(1-C_{3}\right)\left(\sec\left(\frac{\bar{\theta }\pi }{6}\right)-1\right)\right]\left[\sqrt{\frac{1+{C_{1}}^{2}}{3}}\cos\left(\frac{\bar{\theta }\pi }{6}\right)+C_{1}\left(\eta +\frac{1}{3}\sin\left(\frac{\bar{\theta }\pi }{6}\right)\right)\right]\right\}}^{-\frac{1}{n}}$$

where $\bar{\varepsilon_{f}}$ is equivalent fracture strain, *C*_1_, *C*_2_, and *C*_3_ are three parameters to be determined, *n* is the strain component, and *A* is the Swift hardening coefficient.

The criteria for damage evolution play a crucial role in fracture analysis. Under the assumption of monotonic loading conditions, a linear relationship is established between the damage indicator *D* and the equivalent plastic strain ($\bar{\varepsilon_{P}}$). Failure of the element occurs when the specified conditions are satisfied, as shown in Equation (8).

(8) $$D\left(\bar{\varepsilon_{p}}\right)=\int_{0}^{\bar{\varepsilon_{f}}}\frac{d\bar{\varepsilon_{p}}}{d{\bar{\varepsilon }}_{f}\left(\eta ,\;\bar{\theta }\right)}\geq 1$$

## 3. MMC Parameter Calibrations

### 3.1. Experimental Programs

The material used was high-grade X80 pipeline steel plate with a thickness of 10 mm, and the X80 steel plate was produced by Nanjing Iron and Steel Co. (Nanjing, China) The chemical composition of X80 steel plate is summarized in Table 1. The parameters of X80 steel plates are usually constant for a given pipe type. The quasi-static tensile test was conducted using a PLD-50 dynamic fatigue testing machine in displacement control mode, with a loading rate of 0.5 mm/min until the specimen fractured. The test was performed at room temperature. A DH3818Y static strain gauge monitored and recorded real-time strain changes on the specimen’s surface. Given the test conditions and the required measurement accuracy, a 120-3AA resistive strain gauge, manufactured by Shanghai Chengke (Shanghai, China), was selected.

**Figure 2:**
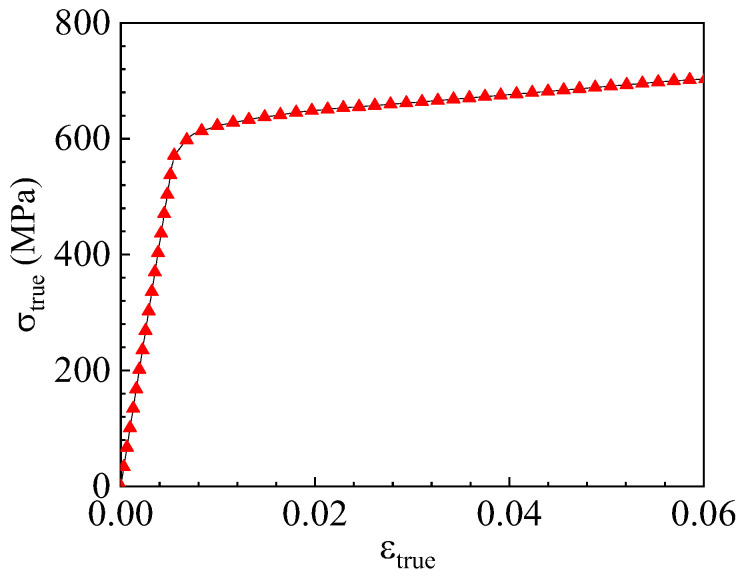
True stress–strain curves for X80 steel.

**Figure 3:**
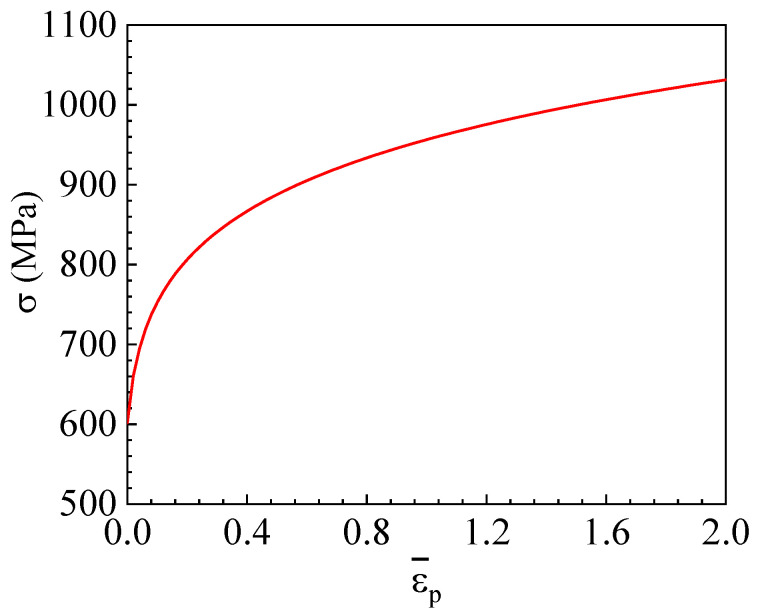
Plastic stress–strain curve fitted using the Swift hardening law.

Figure 2 illustrates the true stress–strain curve obtained during the tests. The graph displays only the segment before a true strain of 0.06 due to the significant necking observed in the specimen beyond this point. Due to the lack of a clear plateau during the yielding phase in X80 steel, as specified by the ASTM E8/E8M [39] standard, the stress corresponding to the residual plastic strain of 0.2% was designated as the yield stress. The material’s plastic behavior can be modeled using the Swift hardening law [40]. The parameters of the Swift law are calibrated to the plastic curve preceding necking, and the plastic curve post-necking is extrapolated from the pre-necking curve. The Swift law is formulated as shown in Equation (9).

(9) $$\bar{\sigma }\left(\bar{\varepsilon_{p}}\right)=A{\left(\bar{\varepsilon_{0}}+\bar{\varepsilon_{p}}\right)}^{n}$$

where $\bar{\varepsilon_{0}}$ is the yield strain. The fitting yielded *A* = 954.9 MPa, $\bar{\varepsilon_{0}}$ = 0.015, and *n* = 0.11. Figure 3 shows the fitted Swift hardening curve.

### 3.2. Specimen Design

**Figure 4:**
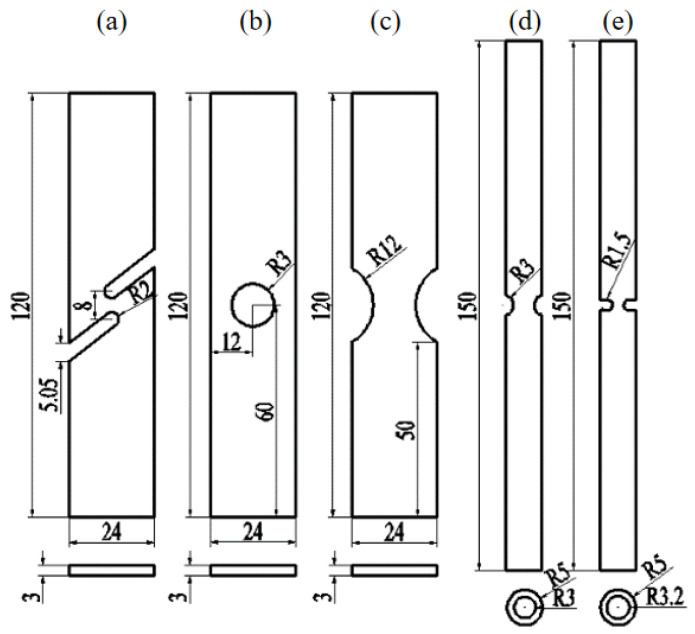
Specimen geometric dimensions (mm): (**a**) FS; (**b**) CH; (**c**) NT; (**d**) NRB3; and (**e**) NRB1.5.

To investigate ductile fracture in materials across a broad spectrum of stress conditions, we devised five specimens: the thin plate shear specimen (FS), center-hole specimen (CH), thin plate notched tensile specimen (NT), and notched round bar tensile specimens with radii of 3 mm (NRB3) and 1.5 mm (NRB1.5). Figure 4 presents the dimensions of these specimens. In Figure 4a, the FS specimen is depicted, exhibiting a stress triaxiality close to 0 at the notch. Figure 4b illustrates the CH specimen, with a stress triaxiality near 0.4 at the notch. Figure 4d,e portray the NRB3 and NRB1.5 specimens, respectively, with stress triaxialities calculated using the Bridgman formula as 0.907 and 1.359, respectively.

**Figure 5:**
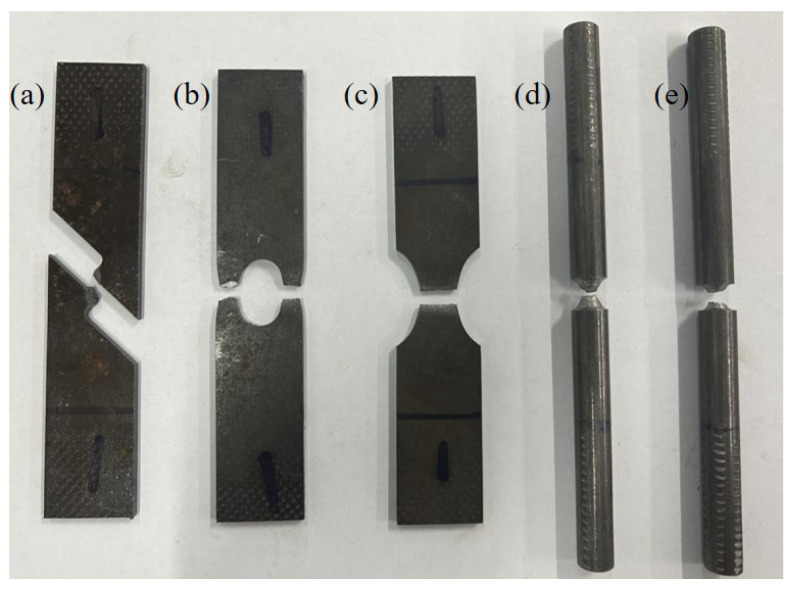
Specimens: (**a**) FS; (**b**) CH; (**c**) NT; (**d**) NRB1.5; and (**e**) NRB3.

Three sets of tensile tests were carried out on each of the five different specimens to ensure the reliability of results. The specimens were subjected to stretching at a loading rate of 0.5 mm/min until fracture transpired. The fractured specimens are depicted in Figure 5.

### 3.3. MMC Parameter Calibration at Failure Initiation

**Figure 6:**
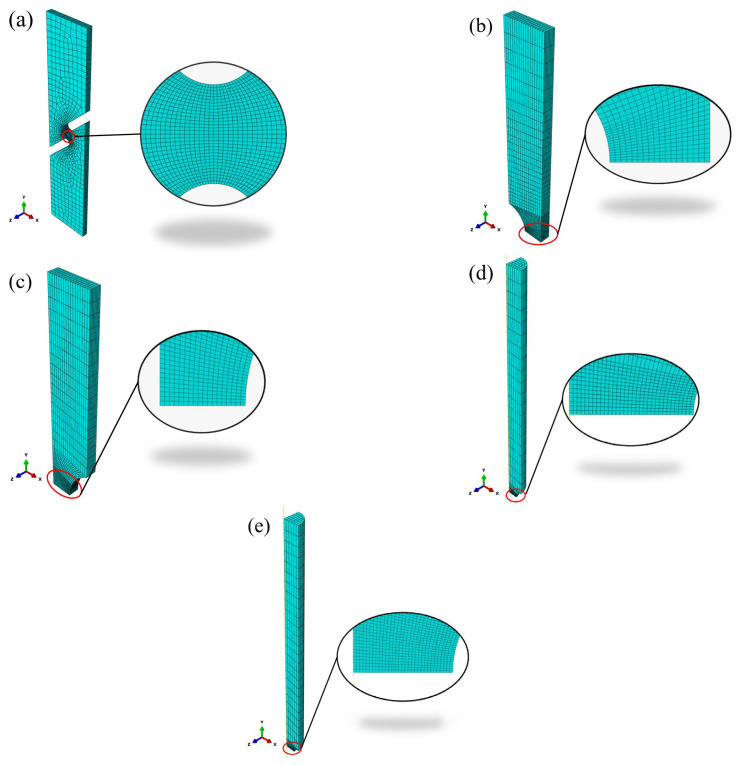
Specimen models and mesh: (**a**) FS; (**b**) CH; (**c**) NT; (**d**) NRB3; and (**e**) NRB1.5.

The finite element software ABAQUS 2022 was utilized to model the specimen with a notch. To simulate crack propagation, the model’s mesh was locally refined at the location where the notch-induced fracture occurred. The finite element model used the 8-node solid reduced integral cell type C3D8R. Models of the five specimen types are depicted in Figure 6.

The mechanical response of all specimens under mono-tonic loading was computed by integrating the VUMAT subroutine, which included only the elastic-plastic principal structures, into the ABAQUS/Explicit solver (Dassault SIMULIA, RI, USA). Displacement loading was applied, and the displacement magnitude corresponded to the value at the point where the test load–displacement curve exhibited a sudden change in the descending section.

Recognizing the symmetry of the specimens, the specimens were modeled using symmetric boundary conditions. For the FS specimen, a half-model was established with ZSYMM symmetry boundary conditions applied in the thickness direction, as shown in Figure 6a. For the CH and NT specimens, a quarter-model was created, applying YSYMM symmetry boundary conditions in the length direction and ZSYMM symmetry boundary conditions in the thickness direction, as illustrated in Figure 6b,c. For the NRB1.5 and NRB3 specimens, an eighth-model was employed, with YSYMM symmetry boundary conditions in the length direction, XSYMM symmetry boundary conditions in the width direction, and ZSYMM symmetry boundary conditions in the thickness direction, as depicted in Figure 6d,e. To simulate crack development, a denser mesh was applied at the notch of each specimen type, while the mesh away from the notch was set sparser. For the FS, CH, and NT specimens, a 0.2 mm mesh was employed in the notched region; for NRB1.5 and NRB3 specimens, a 0.1 mm mesh was applied in the notched region. The boundary conditions included fixing the bottom, displacement loads were applied at the top, and symmetric constraints were applied to the symmetric plane.

**Figure 7:**
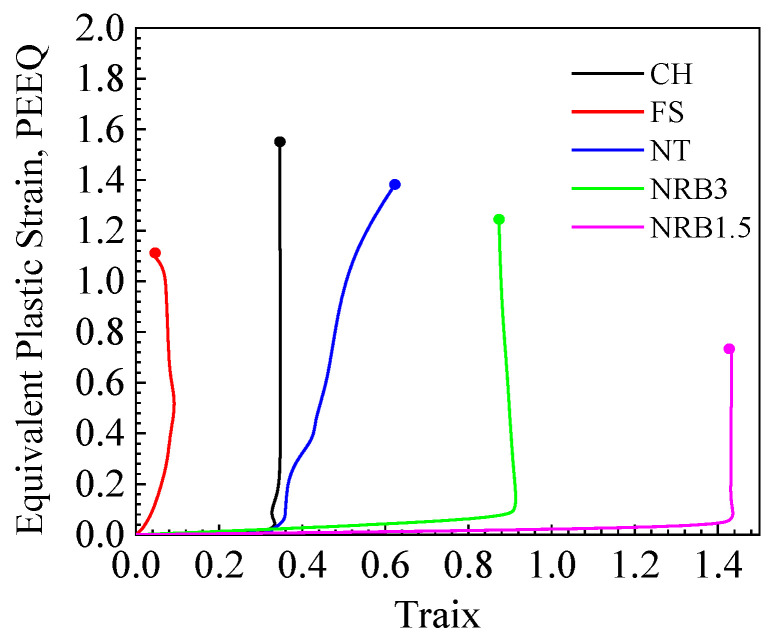
Equivalent plastic strain–stress triaxiality.

**Figure 8:**
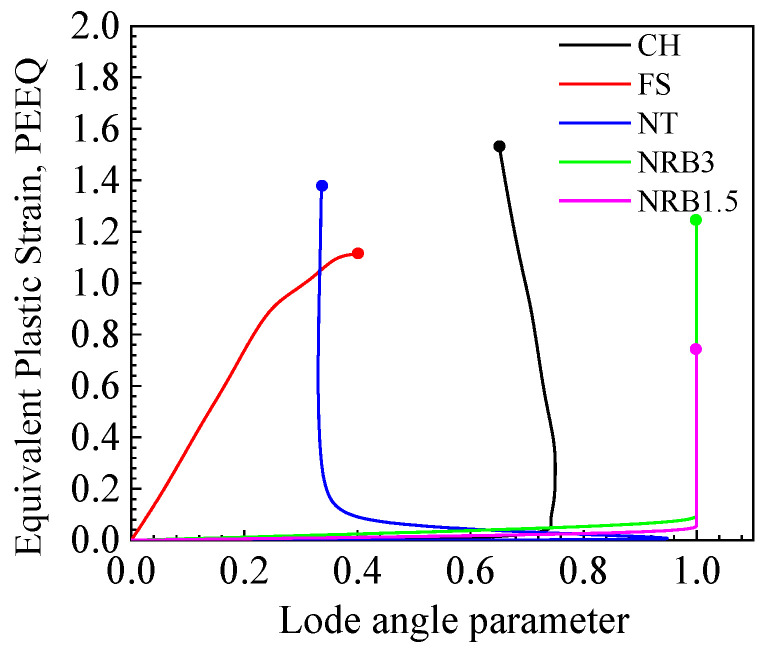
Equivalent plastic strain–Lode angle parameter.

Initially, an undamaged elastoplastic model was created using finite element analysis, and the displacement curves obtained were compared with the experimental results. The failure point was identified by matching the simulation results at the critical displacement, defined as the average displacement of three samples at the point of sudden load drop. After marking the critical displacement for each model, the history of equivalent plastic strain, related to stress triaxiality (*η*) and the Lode angle parameter ($\bar{\theta }$), in the critical elements was obtained, as illustrated in Figure 7 and Figure 8. The circular nodes in the figures denote the onset of fracture. The information was obtained from the integration point of the element with the largest equivalent plastic strain, which was assumed to be where the fracture initiated. For the CH, NRB3, and NRB1.5 specimens, as the equivalent plastic strain increased, the stress triaxiality and Lode angle parameter remained almost constant. Figure 7 shows that the fracture strain does not decrease with increasing stress triaxiality, primarily due to a notable difference in the Lode angle parameter of the FS specimen compared to the others.

Utilizing the mean of sudden load drop points from three experimental sets as the failure initiation point enabled a more precise determination of the onset of failure. Subsequently, the displacement at this juncture aligned with the displacement derived from the finite element simulation curve to pinpoint the initiation point of fracture. Utilizing the equivalent plastic strain, stress triaxiality, and Lode angle parameters at the crack initiation points of the five specimens, the remaining parameters (*C*_1_, *C*_2_, and *C*_3_) of the MMC fracture model were calibrated through the implementation of the MATLAB optimization program. The three unspecified parameters for the MMC model are represented as *C*_1_ = 0.0187, *C*_2_ = 496.56, and *C*_3_ = 0.8864.

### 3.4. Mesh Size Sensitivity Analysis

The mesh size significantly impacts simulation results when using a damage model combined with element deletion to simulate crack propagation. This is because the dissipation of mechanical energy due to element deletion from crack propagation is related to the mesh size. As the mesh size increases, the mechanical energy dissipation (energy release) for each step of crack propagation also increases. For the overall structural behavior, a larger mesh size results in a smoother fracture resistance curve. To investigate the influence of mesh size on the MMC criterion, the CH and NRB3 specimens were selected for mesh sensitivity analysis using the calibrated MMC fracture parameters, with mesh sizes of 0.1 mm, 0.2 mm, 0.4 mm, and 0.6 mm.

**Figure 9:**
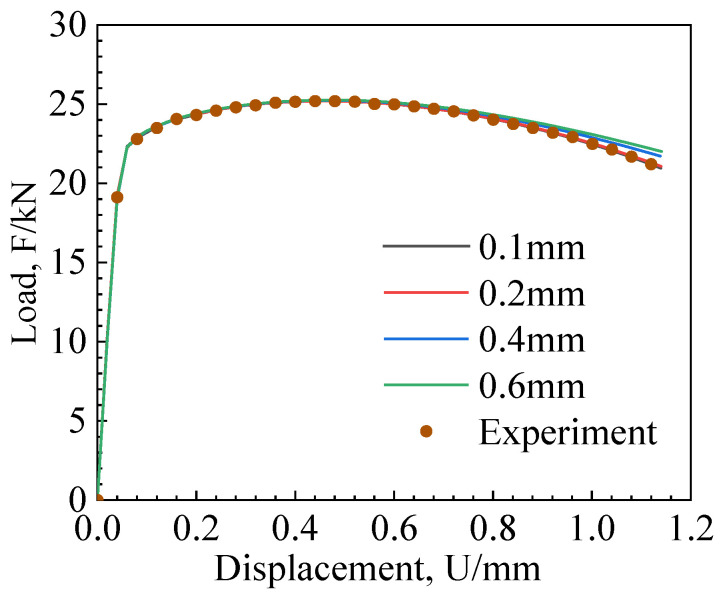
CH specimen mesh size sensitivity analysis.

**Figure 10:**
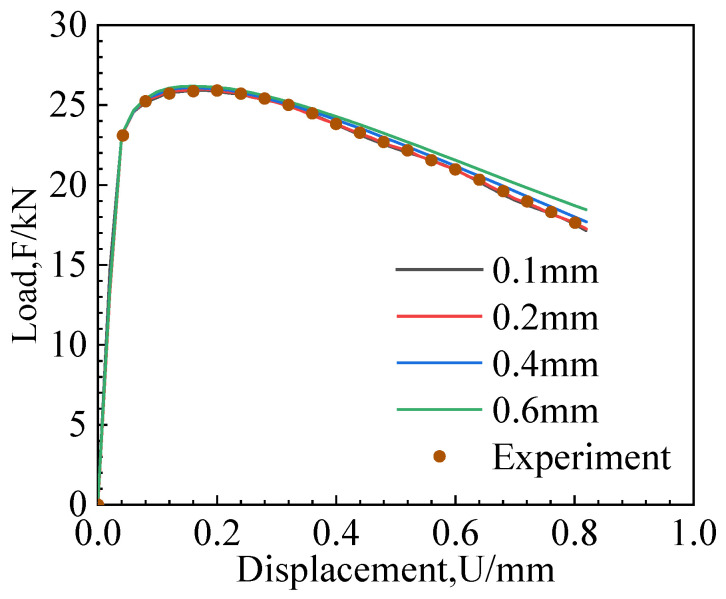
NRB3 specimen mesh size sensitivity analysis.

Figure 9 and Figure 10 show the load–displacement curves for different mesh sizes. In the elastic phase, the load–displacement curves of different mesh sizes coincided. In the plastic phase, larger mesh sizes require greater loads. The experimental load–displacement curve aligns with the curve for a mesh size of 0.2 mm, indicating that the optimal mesh size for the MMC criterion is 0.2 mm.

## 4. Finite Element Program for Pipe Wide Plate

### 4.1. Geometric Dimensions and Boundary Conditions

**Figure 11:**
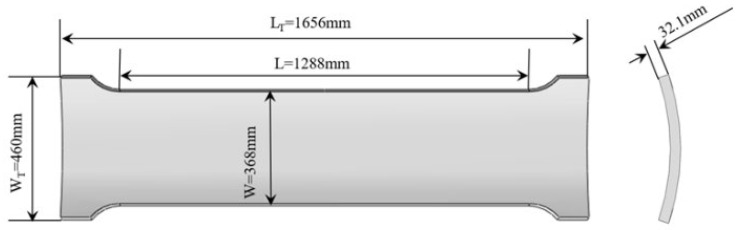
Wide plate dimensions.

Employing ABAQUS 2022 for the analysis of a three-dimensional wide plate model with surface cracks, the dimensions are illustrated in Figure 11. Establishing only a 1/4 model of the wide plate was adequate due to the specimen’s symmetry. Symmetric boundary conditions were implemented in both the circumferential and axial directions for the wide plate model of the pipeline. At the clamped end of the pipe-wide plate, a reference point (RP-1) was designated, and kinematic coupling was implemented between the clamped end and the reference point. Throughout the loading process, a displacement was applied along the axial direction at the reference point RP-1.

### 4.2. Mesh Model

A high level of computational accuracy, a reduction in the number of model elements and nodes, as well as the mitigation of computational costs were strategically achieved through a thorough preliminary investigation. Consequently, the definitive global seed size for the gas pipeline was established at 30 mm. The mesh configuration was predominantly characterized by hexahedral elements, wherein structured meshing was implemented as the primary technique, complemented by swept meshing as a secondary method. The chosen element type aligned with the eight-node reduced integration hexahedral element, corresponding precisely to the C3D8R finite element category.

**Figure 12:**
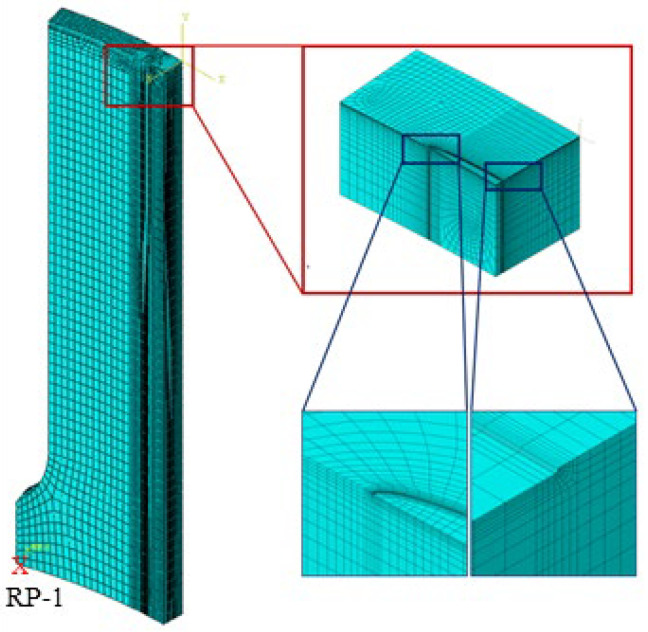
Mesh of the pipeline wide plate.

Within the specific region encompassing the crack, meticulous attention was paid to the definition of the minimum mesh size, precisely set at 0.05 × 0.05 × 0.1 mm, a visual representation of which can be found in Figure 12. The grid in the thickness direction of the cracked surface was set to 0.075 in conjunction with the unit deletion technique feature to maintain consistency with the wide plate crack size. The overall mesh configuration exhibited a maximum size of 4 × 10 × 30 mm, encompassing an approximate total mesh count of 40,000.

## 5. Results and Discussions

### 5.1. Pipe Wide Plate Fracture Process

**Figure 13:**
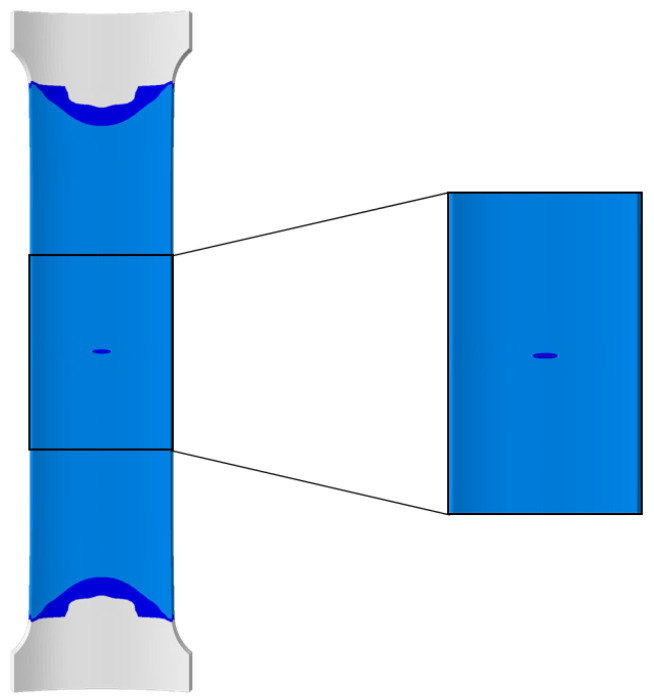
Localized area of pipeline wide plate.

Significant deformation occurs in the central region of the pipeline plate during the fracture process due to its large width. To clearly observe the stress variations in the crack region, the central area with the crack, representing one-third of the plate’s total length, is magnified, as shown in Figure 13.

**Figure 14:**
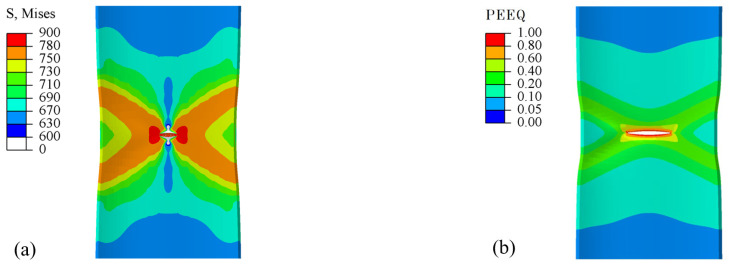

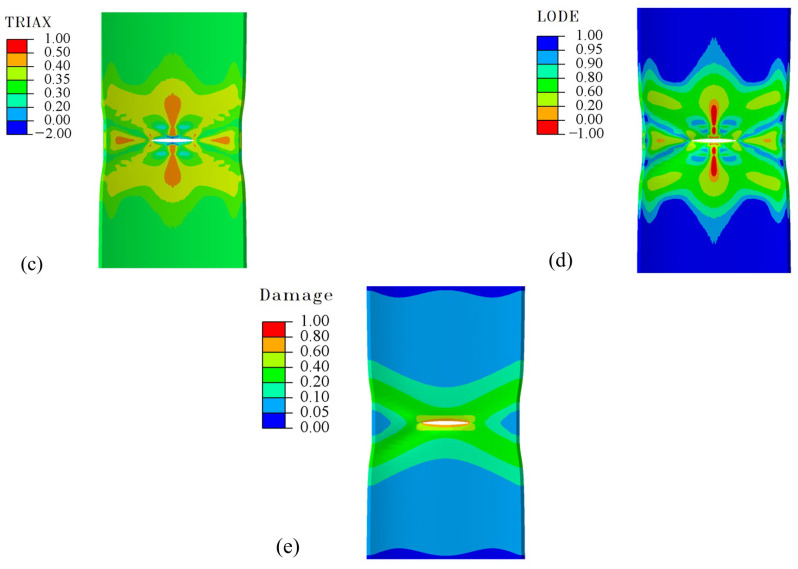
Cloud plots of the wide plate at the moment of crack penetration through the wall thickness: (**a**) Mises; (**b**) PEEQ; (**c**) TRIAX; (**d**) LODE; and (**e**) Damage.

In the tensile testing phase, cloud maps depicting Von Mises (Mises), equivalent plastic strain (PEEQ), stress triaxiality (TRIAX), Lode angle parameter (LODE), and damage value (Damage) play a pivotal role in interpreting experimental outcomes. The selected cloud map pertains to the wide plate of a pipeline with a crack defect, specifically at the instance when the crack breaches the wall thickness, as illustrated in Figure 14. An examination of the figure reveals a notable resemblance in the distributions of PEEQ and Damage. This correspondence stems from the fact that the MMC criterion is grounded in a strain-based approach.

### 5.2. Effect of Crack Shape on the Tensile Properties of Pipeline Wide Plate

In order to study the influence of different crack shapes on the tensile performance of the pipeline wide plate, two common crack shapes, circular and elliptical, were selected. Considering the significant impact of the cross-sectional area of the pipeline wide plate on the load–displacement curve during tensile testing, an elliptical crack with a length of 2*c* = 50 mm and a depth of *a* = 3 mm was chosen. Based on these dimensions, the radius of a circular crack with the same cross-sectional area was calculated to be 8.66 mm.

**Figure 15:**
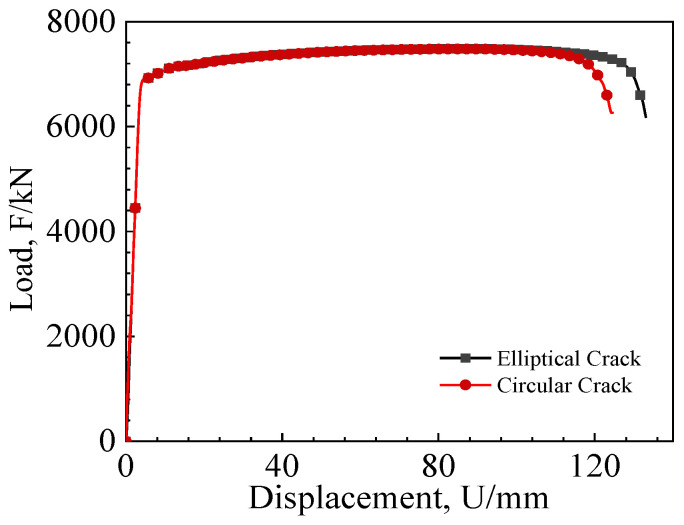
Tensile load–displacement curves of wide plates with different crack shapes.

Figure 15 depicts the load–displacement curves for pipeline wide plates with different crack shapes. From the figure, it can be observed that, before the initiation of the crack, the load–displacement curves for both shapes of cracks completely overlap. However, as the crack begins to propagate, the load–displacement curve for the circular crack starts to drop earlier. By comparison, elliptical cracks demand a greater displacement for penetration through the wide plate. This finding suggests that circular cracks exhibit a diminished load-bearing capacity compared to their elliptical counterparts.

**Figure 16:**
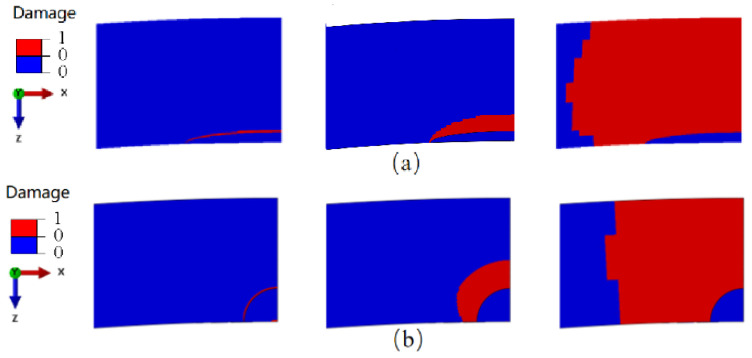
Analysis of crack extension paths for different shapes: (**a**) elliptical crack; (**b**) circular crack.

Figure 16 presents a comparison of the crack propagation paths for different crack shapes at three critical stages: crack initiation, extension to half the thickness of the wide plate, and complete penetration through the wall thickness. Utilizing the MMC criterion and element deletion technique, the damage value of a failed element was set to zero. In the figure, red areas indicate failed regions, while blue areas represent intact regions. The propagation speed in the Z direction (thickness direction) is observed to be greater than that in the X direction (circumferential direction) for elliptical cracks in the wide plate. By contrast, circular cracks exhibit relatively similar propagation speeds in both directions. At the crack initiation stage, the reference point displacements are 32.02 mm for the elliptical crack and 20.18 mm for the circular crack. When the crack extends to half the thickness of the wide plate, these displacements increase to 130.60 mm for the elliptical crack and 117.42 mm for the circular crack. Upon full penetration of the wall thickness, the displacements are 134.94 mm for the elliptical crack and 124.12 mm for the circular crack.

### 5.3. Effect of Surface Crack Size on the Location of Crack Initiation in Wide Plates

**Figure 17:**
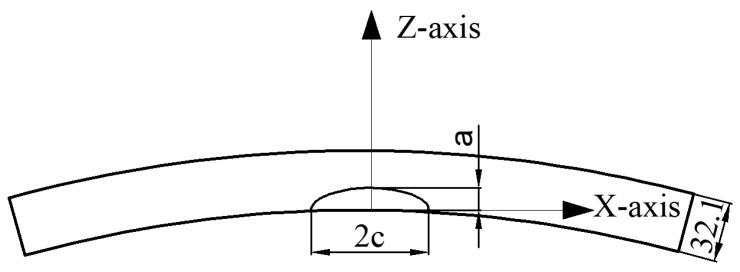
Crack section size.

To comprehensively investigate the propagation behavior of elliptical cracks with diverse shapes on the surface of a pipeline wide plate, finite element models of tension specimens with cracks of varying sizes were established. The geometric and mesh structures of the model aligned with those depicted in the preceding figure. Notably, adjustments were introduced to the initial dimensions of the cracks. The surface crack shape is schematically shown in Figure 17. While maintaining a constant initial crack length of 2*c* = 50 mm, five elliptical cracks with distinct aspect ratios were generated by modifying the crack depth. The five crack depths were specified as *a* = 5 mm, *a* = 10 mm, *a* = 15 mm, *a* = 20 mm, and *a* = 25 mm, respectively.

**Figure 18:**
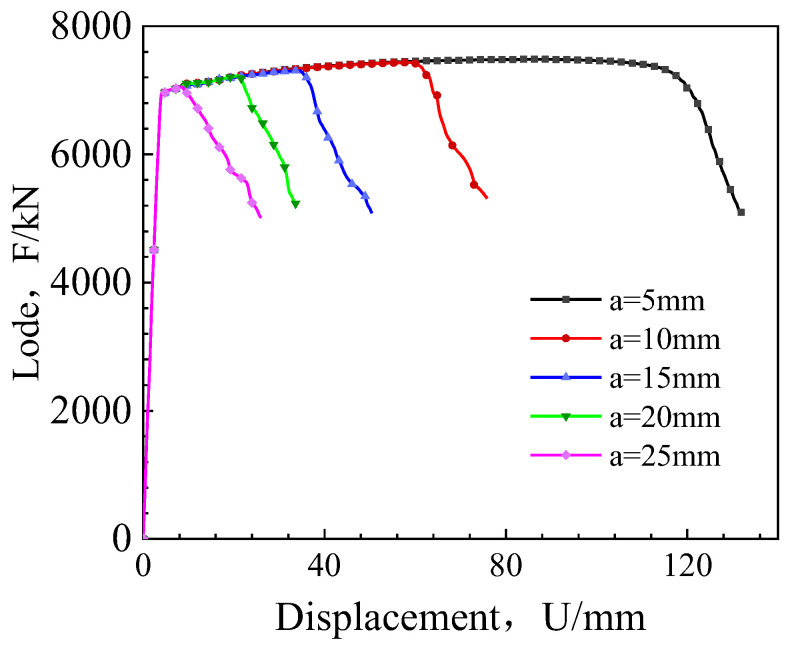
Load–displacement curves for elliptical cracks with different shape ratios.

Figure 18 presents a comparative chart of the load–displacement curves for elliptical cracks with different aspect ratios. Observing the figure reveals that, with an increase in crack depth, the peak load of the pipeline wide plate gradually decreases. However, the rate of descent diminishes as the crack depth increases. Thorough analysis of these curves affords a comprehensive comprehension of the behavioral traits and propagation patterns of cracks exhibiting distinct shapes during the tensile process. This insight, therefore, provides a valuable reference for the safety assessment of materials and structures in engineering applications.

The stress intensity factor (*K*) is a critical parameter that characterizes the intensity of the stress–strain field near a crack tip, thereby determining the rate of crack propagation. A higher stress intensity factor increases both the likelihood and speed of crack growth. This factor is influenced by several variables, including the applied load, the number, length, and location of cracks, as well as the geometry of the material. Among these, crack depth (*a*) significantly affects *K*, with deeper cracks leading to greater stress concentration at the crack tip, thereby increasing the value of *K*. The formula for calculating the stress intensity factor is provided in Equation (10).

(10) $$K=\sigma \sqrt{\pi a}\cdot g\left(\frac{a}{t},\frac{c}{t}\right)$$

where σ is the applied stress, *a* is the crack depth, *c* is the crack length, *t* is the thickness of the wide plate, and $g\left(\frac{a}{t},\frac{c}{t}\right)$ is a function related to the crack length, depth, and the geometry of the specimen.

**Figure 19:**
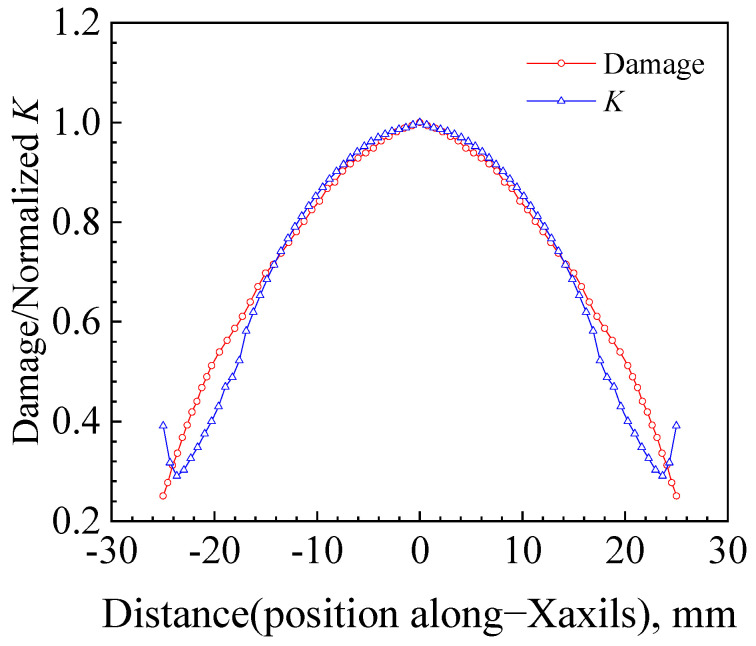
A comparison of damage values obtained using the MMC criterion with the *K* profile along the entire crack front.

The influence of surface crack shape evolution on the displacement of the maximum stress intensity factor (*K*) or maximum damage value location along the crack front remains uncertain. To better understand the relationship between the crack growth driving force and crack shape, and to potentially reduce unnecessary conservatism in structural design and maintenance, it is beneficial to study the evolution of crack shape through the plate thickness in more detail. This study employed numerical simulations to investigate the detailed profile of the crack driving force along the entire crack front as the crack shape changed, including its deepest and surface points, as well as the maximum damage value that may move between these points. An elliptical crack with a length of 2*c* = 50 and a depth of *a* = 5 was selected. The damage value distribution along the crack front obtained using the MMC criterion was compared with the *K* distribution obtained using static calculations, as shown in Figure 19. Figure 19 demonstrates that the value at the crack surface point undergoes a sudden change when using *K* calculation, whereas the MMC criterion accurately calculates the detailed profile of the driving force along the entire crack front. The distribution trends of damage values at the crack front and *K* along the X-axis in Figure 19 closely aligned with the experimental results reported by Wang et al. [32].

**Figure 20:**
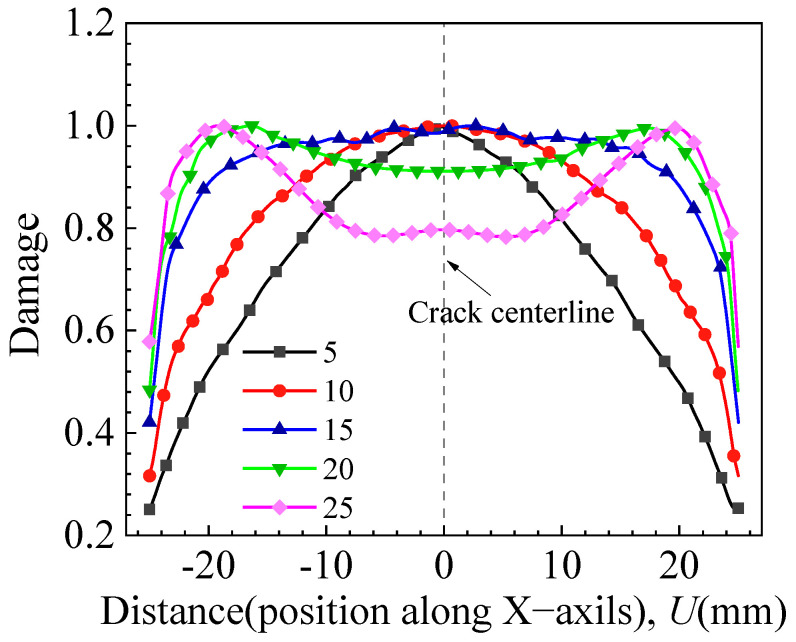
Distribution of damage values along the X-axis for different crack shapes.

In Figure 20, the distribution of damage values along the X-axis at the moment of crack initiation is presented for various crack shapes. From the figure, it is evident that a decrease in the crack shape ratio (*a*/*c*) corresponds to an increased difference in damage value distribution along the crack front. Concurrently, damage values near the inner side of the wide plate along the crack front rise with the growth of the crack shape ratio (*a*/*c*). Notably, when the crack depth (*a*) equals half the thickness of the wide plate, the damage values reach a plateau. Thus, when the crack depth exceeds half the thickness, the maximum damage value is observed near the inner side of the wide plate along the crack. When the crack depth is less than half the thickness, the maximum damage point is positioned in the middle of the elliptical crack front.

**Figure 21:**
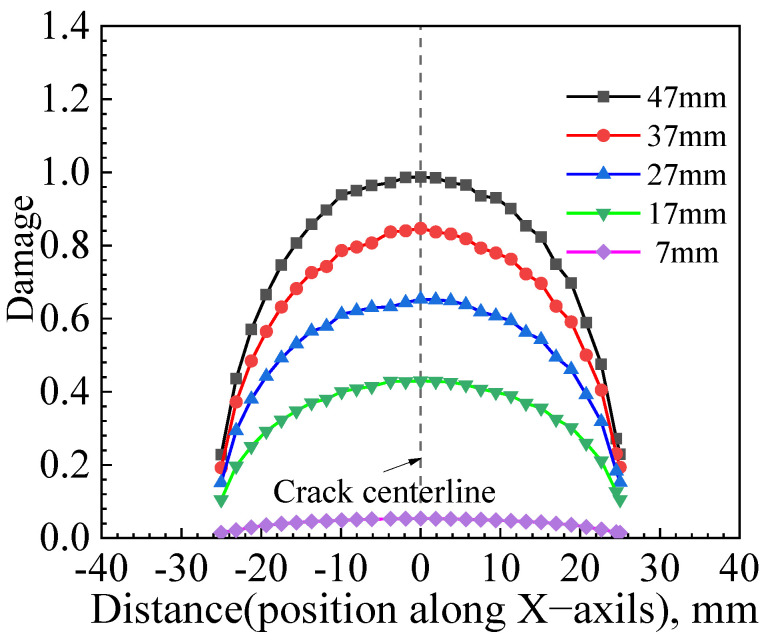
Distribution of damage values of elliptical cracks (*a*/*c* = 1/5) along the X-axis at different tensile displacements.

Figure 21 shows the distribution of damage values along the X-axis direction along the crack front before crack initiation at five different moments, with a crack length of 2*c* = 50 mm and a crack depth of *a* = 5 mm. These moments correspond to displacements at reference points of u = 47 mm, u = 37 mm, u = 27 mm, u = 17 mm, and u = 7 mm, respectively. The figure shows that during the cumulative damage process along the crack front, the point with the maximum accumulated damage for the elliptical crack with a depth of *a* = 5 mm is at the middle of the crack front. This suggests that the crack is likely to initiate here. During the first 10 mm displacement increment, the damage value increment is the highest, and with subsequent increments of the same displacement, the increment in damage values gradually decreases.

**Figure 22:**
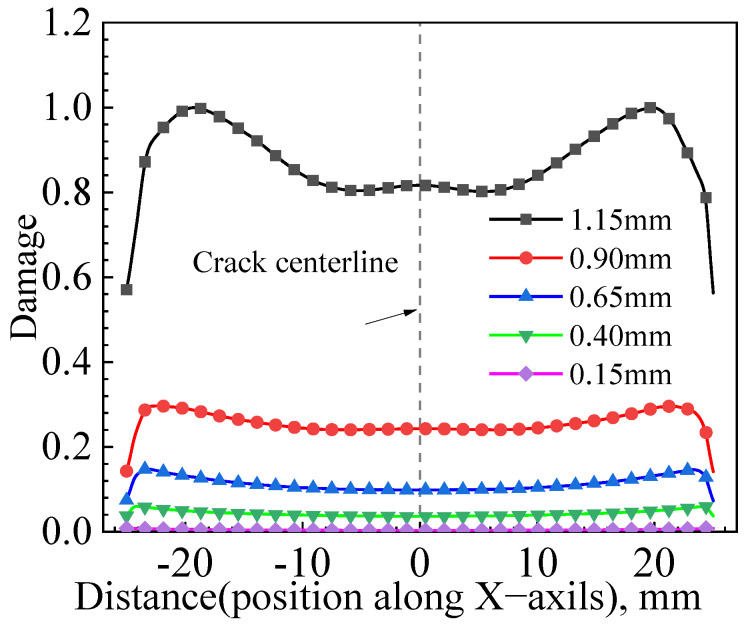
Distribution of damage values of circular cracks (*a*/*c* = 1) along the X-axis at different tensile displacements.

Figure 22 shows the distribution of damage values along the X-axis direction along the crack front before the initiation of a circular crack, with a crack length of 2*c* = 50 mm and a crack depth of *a* = 25 mm. These moments correspond to displacements at reference points of u = 1.15 mm, u = 0.90 mm, u = 0.65 mm, u = 0.40 mm, and u = 0.15 mm, respectively. The figure shows that during the cumulative damage process along the crack front, the point with the maximum accumulated damage for the circular crack with a depth of *a* = 25 mm is near the inner side of the wide plate. This suggests that the crack is likely to initiate here. During the first 0.25 mm displacement increment, the damage value increment is minimal, and with subsequent increments of the same displacement, the damage value increment gradually increases.

## 6. Conclusions

This study employed the MMC ductile fracture criterion to analyze the impact of various crack shapes on the fracture behavior of oil pipelines. The main conclusions of this research are as follows:

(1) Fracture performance experiments were conducted under monotonic loading, coupled with finite element analysis. Using a phenomenological hybrid numerical-experimental calibration method, the fracture parameters of the MMC model were established as *C*_1_ = 0.0187, *C*_2_ = 496.56, and *C*_3_ = 0.8864.
(2) At the critical section with equivalent cross-sectional areas, circular cracks exhibited an earlier sudden load decrease compared to elliptical cracks, indicating a reduced load-bearing capacity for circular cracks. Elliptical cracks predominantly extended in the thickness direction, whereas circular cracks demonstrated relatively consistent propagation distances in various directions.
(3) With a constant crack length, as the crack depth increased, the displacement at the point of the sudden load drop gradually diminished. In the cumulative damage process at the crack front, the elliptical crack front with a depth less than half of the wall thickness (*a* = 5 mm) exhibited the highest damage accumulation at its midpoint. Conversely, for the circular crack front with a depth greater than half of the wall thickness (*a* = 25 mm), the highest damage accumulation occurred near the inner measurement point of the wide plate. Additionally, under uniform displacement increment before crack initiation, the damage increment of the elliptical crack with *a* = 5 mm gradually decreased, whereas the increment value of the circular crack with *a* = 25 mm gradually increased.

This study considered only the structural and mechanical effects on fracture behavior, without accounting for the influence of the pipeline’s actual operating environment on the calibration of MMC fracture parameters. Future studies combining these factors may provide a more comprehensive explanation. This study provides valuable guidance for expensive full-scale testing and offers theoretical insight for the design and safety assessment of long-distance oil and gas pipelines.

## Figures and Tables

**Table 1:** Chemical composition of X80 steel plate.

Element	C	Si	Mn	P	S	Cr
wt%	0.047	0.26	1.70	0.01	0.001	0.24

## Data Availability

The raw data supporting the conclusions of this article will be made available by the authors upon request.
